# Methodologies for Pre-Validation of Biofilters and Wetlands for Stormwater Treatment

**DOI:** 10.1371/journal.pone.0125979

**Published:** 2015-05-08

**Authors:** Kefeng Zhang, Anja Randelovic, Larissa M. Aguiar, Declan Page, David T. McCarthy, Ana Deletic

**Affiliations:** 1 Monash Water for Liveability, Department of Civil Engineering, Monash University, Melbourne, VIC, Australia; 2 CRC for Water Sensitive Cities, Melbourne, VIC, Australia; 3 Faculty of Civil Engineering, University of Belgrade, Belgrade, Serbia; 4 CSIRO Land and Water Research Flagship, Waite Laboratories, Adelaide SA, Australia; 5 Environmental and Public Health Microbiology Laboratory, Department of Civil Engineering, Monash University, Melbourne, VIC, Australia; NERC Centre for Ecology & Hydrology, UNITED KINGDOM

## Abstract

**Background:**

Water Sensitive Urban Design (WSUD) systems are frequently used as part of a stormwater harvesting treatment trains (e.g. biofilters (bio-retentions and rain-gardens) and wetlands). However, validation frameworks for such systems do not exist, limiting their adoption for end-uses such as drinking water. The first stage in the validation framework is pre-validation, which prepares information for further validation monitoring.

**Objectives:**

A pre-validation roadmap, consisting of five steps, is suggested in this paper. Detailed methods for investigating target micropollutants in stormwater, and determining challenge conditions for biofilters and wetlands, are provided.

**Methods:**

A literature review was undertaken to identify and quantify micropollutants in stormwater. MUSIC V5.1 was utilized to simulate the behaviour of the systems based on 30-year rainfall data in three distinct climate zones; outputs were evaluated to identify the threshold of operational variables, including length of dry periods (LDPs) and volume of water treated per event.

**Results:**

The paper highlights that a number of micropollutants were found in stormwater at levels above various worldwide drinking water guidelines (eight pesticides, benzene, benzo(a)pyrene, pentachlorophenol, di-(2-ethylhexyl)-phthalate and a total of polychlorinated biphenyls). The 95^th^ percentile LDPs was exponentially related to system design area while the 5^th^ percentile length of dry periods remained within short durations (i.e. 2–8 hours). 95^th^ percentile volume of water treated per event was exponentially related to system design area as a percentage of an impervious catchment area.

**Conclusions:**

The out-comings of this study show that pre-validation could be completed through a roadmap consisting of a series of steps; this will help in the validation of stormwater treatment systems.

## Introduction

Stormwater is increasingly recognized as a valuable alternative water resource [1]. In Australia, treated stormwater is applied mainly to outdoor non-potable uses (e.g. irrigation) and indoor non-potable uses (e.g. toilet flushing and laundry) [2]. Furthermore, stormwater has been used for outdoor, domestic and municipal irrigation purposes in USA, UK and Sweden [1, 3, 4]. In Singapore, there are also examples of harvesting stormwater for potable use via drinking water reservoirs [5]. Indeed, the potential for harvesting stormwater to potable water use is considerable; for example, it is estimated that in Melbourne, Australia, around 400 GL/yr of stormwater runs off the urban catchment, which is roughly equivalent to the amount of potable water currently consumed in the same city [6].

Water Sensitive Urban Design (WSUD) technologies, such as biofilters (rain-gardens and bioretentions) and wetlands, are promising stormwater treatment systems that can reduce the high level of stormwater variability to predictable, manageable levels [7]. They are both soil-based natural systems that provide treatment through a combination of physical (sedimentation, mechanical straining), chemical (sorption) and biological process (plants and microbial uptake) [8]. The filter media, the plants and the configuration of these systems could be carefully chosen to enhance the treatment of pollutants in stormwater [9]. They have been proven to efficiently treat sediments, metals, and nutrients [10, 11], microorganisms [12], and micropollutants [13]. However, these systems are not given any credit for their removal performance when used for stormwater harvesting schemes for almost any end-use. This is mainly due to lack of any methodology on how these systems should be **validated** before they are allowed to become an integral part of a stormwater treatment train for human consumption.

Water treatment validation is the process of ensuring that (i) a treatment system can produce water of the required quality under a defined range of operational conditions, and (ii) it can be monitored in real time to provide assurance that water quality objectives are being continuously met [14]. There are validation frameworks developed for highly engineered water treatment systems for pathogen removal, such as membrane filtration [15], ultraviolet (UV) disinfection [16], activated sludge process and media filtration [14]. However, there are no published guidelines for the validation of WSUD stormwater harvesting systems. The direct application of frameworks developed for highly engineered systems to WSUD stormwater harvesting systems is not possible, because *in-situ* style challenge tests are usually not applicable to big WSUD systems.

This study presents development of a framework for validation of WSUD systems for stormwater harvesting. As with other validation methodology, (e.g. the one proposed by the Department of Health, Victoria (DHV) [14]), the proposed framework contains three stages: (i) Pre-validation, (ii) Validation monitoring, and (iii) Operational monitoring. While parallel work is progressing on development of Stages 2 and 3 (Validation monitoring and Operational monitoring [13]), this paper focuses on Stage 1: Pre-validation. It is the first and very important stage in which the following should be identified: (1) target pollutants, (2) treatment targets, (3) potential removal mechanisms, (4) potential surrogates and (5) operational/challenge conditions. The parameters are directly linked to the end-use of the treated stormwater and treatment system design; for example while for restricted irrigation only sediments and some heavy metals should be considered [17], for treatment to potable standards all pollutants should be considered with a strong emphasis on removal of pathogens and micropollutants. However, while general stormwater quality has been extensively reported in literature [18–20], the knowledge on both pathogens and organic micropollutants in stormwater is limited. In addition, the complex operational conditions of WSUD systems, which are crucial for validation monitoring, have never been examined.

The aim of this paper is to describe the development of the Pre-validation stage of the Validation Framework for stormwater treatment systems by providing: (i) the roadmap of pre-validation procedure, and (ii) methodologies for completing the pre-validation procedure for micropollutant removal by stormwater biofilters and wetlands, when used in treatment to potable water standards. Specific focus is given to the key research gaps: the identification of the target micropollutants and development of the methodologies for identifying the operational and challenge conditions for the selected WSUD systems. While this study focuses only on biofilter and wetlands, micropollutants as the target pollutant, and treatment to potable standards, the developed pre-validation methodologies are general and could be extended to encompass other WSUD systems, pollutants (e.g. metals and pathogens) and end-uses (e.g. in-door non-potable uses). It is the first attempt in literature to provide a robust framework for the validation of natural treatment systems engaged in stormwater harvesting which, if adopted in practice, could support widespread implementation of stormwater harvesting.

## Proposed validation framework for WSUD systems

Table 1 outlines the proposed validation framework for WSUD stormwater treatment systems. The main concepts have been derived from the procedures applied to validation of wastewater recycling systems for non-potable uses [14].

**Table 1:** Proposed validation framework for WSUD stormwater harvesting systems.

*Aims and objectives*		
• The system can produce water of required quality under a defined set of operational conditions	• The water quality objectives are being continuously met under a defined set of operational conditions	• Applicable to a wide range of WSUD systems and sizes
**Pre-validation**	**Validation Monitoring**	**Operational Monitoring**
• Identification of target pollutants in stormwater	• Validation of hydraulics	• Monitoring of the verified surrogates (or directly measuring target hazards)
• Specification of treatment targets	✓ In-situ tracer tests	• Identification of the need for re-validation
• Identification of the potential removal mechanisms and influential factors	✓ Modelling	
• Identification of surrogates and for operational monitoring	• Validation of treatment performance (i.e. removal processes)	
• Establishment of the operational and challenge conditions for systems	✓ Challenge tests—if possible	
	✓ Modelling/lab/in-situ measurements	
	• Verifying relationships between surrogates and pollutants for operational conditions	

### Stage 1: Pre-Validation

Stage 1: Pre-Validation pertains to gathering necessary information for the next two stages, i.e. Validation monitoring and Operational monitoring. It initially includes selection of target pollutants, corresponding treatment mechanisms and targets. Additionally, the challenge conditions need to be determined, such as maximum loading rates, inflow pollutant levels and challenge hydrological regimes (e.g. challenge treatment flow-rate, event volume, duration of dry-wet periods, etc.). Table 2 outlines the proposed roadmap of the Pre-validation stage, consisting of five steps.

**Table 2:** Roadmap for Stage 1: Pre-validation.

Steps	Description	General methods
1. Identify target pollutants in stormwater	Target pollutants is the subject of validation study, and their operational and challenge concentrations in stormwater need to be identified	Catchment audit, monitoring of actual stormwater, and available data on quality of stormwater. Basic statistical analysis of the collected data. 95^th^ percentile concentrations of the data collected should be used as challenge concentrations.
2. Specify the treatment target	The treatment target defines the treatment target that a validated system must provide	Depending on the end use the treatment target will be derived as per relevant guidelines values.
3. Identify removal mechanisms and the influential factors	Successful validation of a treatment process relies upon an understanding of the mechanisms (including influential factors)	Literature review on the properties of the target pollutants, including the treatment process to be validated and the factors that influence the processes.
4. Identify potential surrogate parameters	Continuous monitoring of reliable surrogate is important to provide assurance that the system is under control.	Literature review on potential surrogates of the target pollutants for different processes.
5. Identify operational and challenge conditions for the systems	Operational condition sets the boundaries for which the validation will be accepted, including	Collect local climate data and then determine the operational/challenge conditions based on hydrological modelling and statistical analyses.
	- Treated volume per event	
	- Environmental conditions, including temperature, length of dry period	
	- Flow-rate	

### Stage 2: Validation Monitoring

Stage 2: Validation Monitoring determines the system performance under challenge conditions in order to prove that it can cope in extreme situations. Both hydraulics and treatment performance must be validated, which is conducted using *in-situ* challenge tests. During this step, it is essential that selected surrogates are confirmed using both laboratory and field studies, so that they can be used successfully during operational monitoring. See more details on this stage in Zhang et al [13].

### Stage 3: Operational Monitoring

Stage 3: Operational Monitoring ensures that defined treatment targets are being continuously met during normal operation, by either monitoring pollutants directly or using suitable surrogates. This stage is still to be developed.

## Methods

As already outlined, this study focuses on the Pre-validation stage, with specific focuses on identification of target micropollutants in stormwater (Step 1; Table 2) and identification of operational and challenge conditions for stormwater biofilters and wetlands (Step 5; Table 2) based on the current knowledge gaps in literature. As the operational conditions are system and site specific, the paper selects wetlands and biofilters of different designs and three different climates as examples. While Step 1 and 5 are the main focus of this study, other three steps are also discussed to provide a complete pre-validation roadmap. Step 2 (treatment target) is based on Step 1, whereas Step 3 entails potential removal mechanisms of micropollutants that have been studied extensively in relative natural systems [21–30]. Although there are also knowledge gaps regarding surrogate parameters in natural systems, this study identifies only the potential surrogate parameters for further testing during the later validation monitoring stage.

### Step 1: Identification of target micropollutant in stormwater

**Fig 1:**
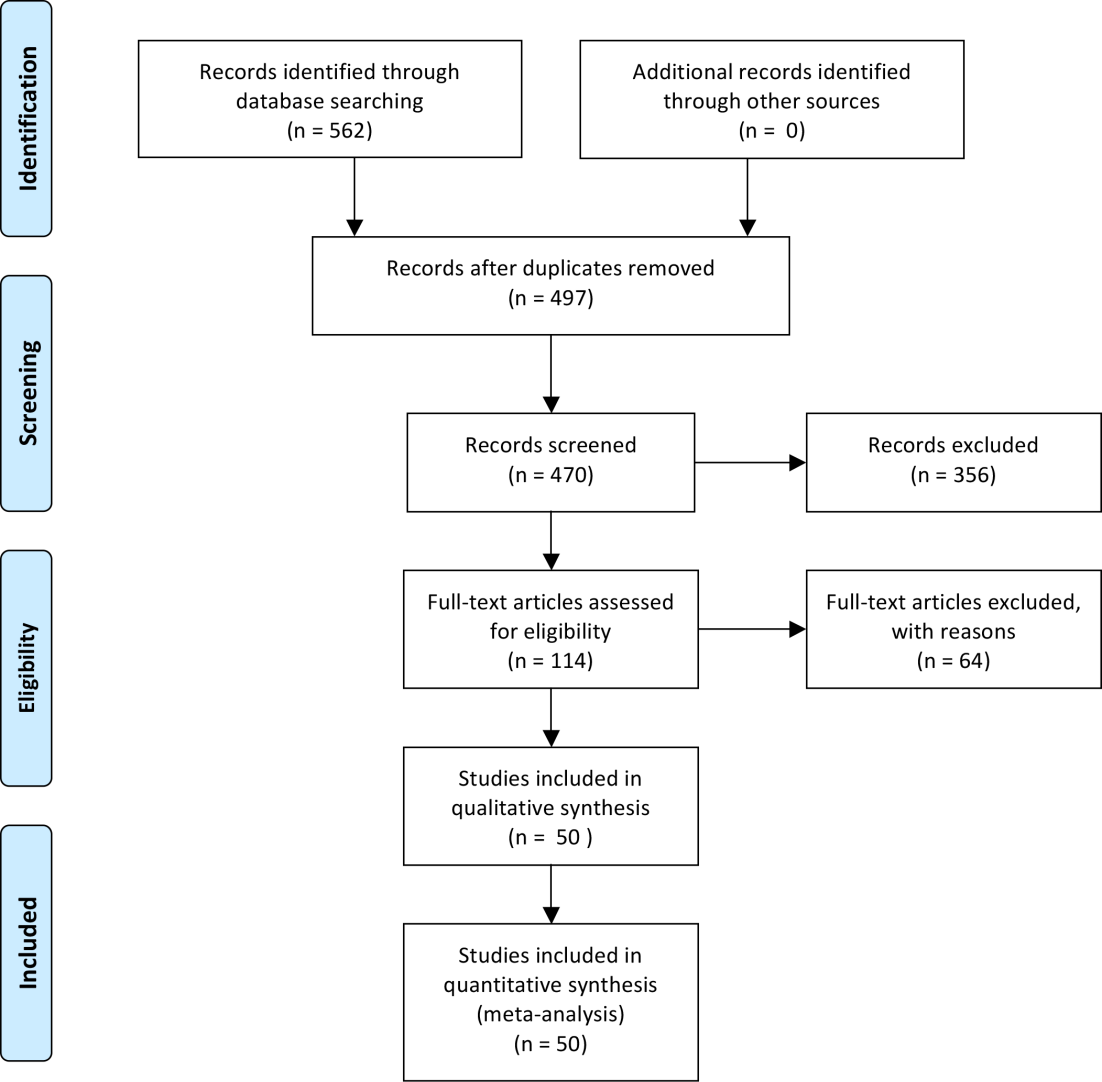
Flow diagram depicting systematic review search results.

A literature review was performed to identify micropollutants in stormwater published over last 30 years. Literature search was undertaken through Monash Library that includes key resources (e.g. Scopus, Web of Science, etc.). The following keywords were utilized: stormwater, micropollutant, pesticide, hydrocarbon, phthalate, polychlorinated biphenyl, phenol, halogenated aliphatic, as well as some specific organic compounds based upon the first search. Reference lists of all included articles were manually searched to identify additional sources of data, missing articles, or meeting abstracts. A primary search yielded 562 studies underwent initial abstract review (Fig 1), of which 445 studies were excluded. Of the remaining 114 studies, only 50 reported the micropollutant concentrations in stormwater and were included in the analysis (Table 3).

**Table 3:** 5^th^, 50^th^ and 95^th^ percentile concentrations of micropollutant interpolated from literature and the corresponding drinking water guidelines: Australia [33]; USEPA [35], WHO [34] and EU[36].

Parameters	n*	5th	50th	95th	Australia	USEPA	WHO	EU
***Pesticides (μg/L)*** [18, 53–70]								
Glyphosate	34	6	70	200	1000	700		
AMPA	26	0.6	3	7				0.1 for
Simazine	32	0.1	0.5	1	20	4	2	each
Atrazine	27	0.3	1	3	20	3	100	pesticide
Diuron	30	0.5	3	8	20			and
Isoproturon	20	< LOD	0.1	0.1			9	0.5 for total
Aldrin	21	0.1	0.6	2	0.3 total		0.3 total	
Dieldrin	19	0.1	0.4	1				
***Phthalates (μg/L)*** [18, 54, 56, 65, 67–75]								
Di-(2-ethylhexyl)-phthalate	60	4	20	50	10	6	8	
Dibutyl phthalate	27	0.5	4	10				
Benzyl butyl phthalate	18	0.7	6	20				
Di-n-octyl phthalate	26	0.3	3	8				
***Phenols (μg/L)*** [18, 53, 54, 56, 67–71, 73–81]								
Phenol	16	6	50	100				
Pentachlorophenol	19	3	30	90	10	1	9	
Nonylphenol	36	0.5	3	7				
***Polychlorinated biphenyls (PCBs) (μg/L)*** [18, 56, 68–70, 77, 82, 83]								
Total PCBs	34	0.1	0.4	0.9		0.5		
***Halogenated aliphatics (μg/L)*** [18, 54, 56, 62, 70, 74, 84, 85]								
Chloroform	23	0.5	4	9		80	300	100
***Monocyclic aromatics (μg/L)*** [18, 54, 56, 62, 70, 73, 74, 84, 86–88]								
Benzene	30	0.5	4	10	1	5	10	1
Ethylbenzene	21	0.4	2	6	300	700	300	
Toluene	23	0.5	4	10	800	1000	700	
***Polycyclic aromatic hydrocarbons (PAHs) (μg/L)*** [18–20, 54, 56, 61, 67–70, 73–76, 87–98]								
Total PAHs	117	40	400	1000				
Naphthalene	60	0.2	2	4				
Acenaphthylene	39	0.01	0.05	0.1				
Acenaphthene	36	0.02	0.06	0.1				
Fluorene	42	0.05	0.3	0.8				
Phenanthrene	48	0.7	8	20				
Anthracene	39	0.3	3	8				
Fluoranthene	58	5	60	100				
Pyrene	56	4	40	100				
Benzo(a)anthracene	52	0.3	3	8				
Chrysene	46	0.6	5	10				
Benzo(a)pyrene	43	0.3	3	8	0.01	0.2	0.7	0.01
Dibenz(a,h)anthracene	43	0.1	1	4				
Benzo(b)fluoranthene	40	0.6	5	10				
Benzo(k)flouranthene	40	0.3	3	8				
Benzo(g,h,i)perylene	44	0.1	0.5	1				
Indeno(1,2,3-cd)pyrene	43	0.4	4	10				
	***Stormwater parameters (mg/L)*** * ***From NRMMC-EPHC-NHMRC [8]***							
pH		5	6	7.3				
Suspend solids		19	77	254				
Total nitrogen		0.6	3	7				
Total phosphorus		0.1	0.4	1				
Biochemical oxygen demand		7	43	141				
Chemical oxygen demand		33	56	9				

* Number of data points used for estimation.

Data reported in [54, 60, 80, 89, 90] are based on discrete/grab samples, while the rest are based on event mean concentration (EMC).

Various types of micropollutant concentration statistics were presented. Event Mean Concentrations (EMC), representing the flow-weighted average concentration of a single runoff event, are the most representative values for characterisation of stormwater, and thus most commonly reported (Table 3). All EMC values that were found in literature were included in our statistical analysis, even a single value for a given site. If statistics of EMC values were given (e.g. maximum, minimum, medium, etc.), these values were taken. It should be noted that the distribution of EMC values in each piece of literature is seldom indicated. Maximum and minimum concentrations of discrete samples recorded in a single catchment were also considered, since extremes are important for determination of challenge conditions. Only studies which contained greater than 5 rainfall events per site were included; in these cases, the minimum, median and maximum values of the concentrations were estimated. If only the range of discrete concentrations was available (and based on a measurement campaign that had more than 5 events per site), minimum and maximum concentrations were assumed to correspond with the range.

The reviewed papers indicated that >100 micropollutants were identified in stormwater. A micropollutant was selected only if it was measured at more than three catchments. After the screening, data on 37 stormwater micropollutants (Table 3) was used to create the data set.

Due to the scarcity and variability (gathered from various catchments and climates around the world), a triangular distribution was developed for each selected micropollutant. The triangular distribution is a continuous probability distribution with a probability density function shaped like a triangle. It is defined by three values: the minimum value, the maximum value and a mode value (most likely value). This distribution has also been adopted in other studies due to data scarcity [31, 32]. Catchment specific concentrations were not discussed due to the relative limited studies on the micropollutants in stormwater. The absolute minimum and maximum concentrations within the data set were selected as minimum and maximum values for each micropollutant. In cases when the minimum is below detection limit, zero was used; the mode value was determined by establishing the median value among all the median values collected, hence equates with the median of the triangular distribution. The 5^th^, 50^th^ and 95^th^ percentile concentrations were then established based on the triangular distribution developed. The 95^th^ percentile concentration was selected as challenge concentration, since it is recommended as challenge concentrations in validation monitoring of other water types [14]. The raw data underlying the statistics of micropollutants in stormwater could be found in S1 Table.

### Step 2: Specification of the treatment targets

Worldwide drinking water guidelines values [33–36] were sourced to set the treatment targets; these guidelines were used as only drinking water guidelines define specific values for a wide range of micropollutants for human safety protection, while other standards (e.g. irrigation guidelines, [37]) do not indicate values for micropollutants or they report higher values that may not pose a threat to humans. The targets set by the Australian Guidelines for Water Recycling: Stormwater Harvesting and Reuse [8] were also used in the study (Table 3).

### Step 3: Identification of removal mechanisms and the influential factors

A literature review was carried out to identify potential micropollutants removal mechanisms for different micropollutants groups identified in Step 1. Removal mechanisms of micropollutant have been extensively reported in wetlands [21–26] stormwater bioretention systems [27, 28] and related soil-based systems such as aquifers [29, 30]. These removal processes are largely dependent on the physical-chemical properties of the micropollutants, therefore the latter were also reviewed [38].

### Step 4: Identification potential surrogate parameters

Identification of potential surrogates parameters was conducted by reviewing the current literature on the surrogates for micropollutants during different treatment systems, such as aquifer recharge systems [39], chemical oxidation [40, 41] and ozonation [42], as no studies on surrogates have been reported for stormwater biofilters and wetlands.

### Step 5: Determination of operational and challenge conditions for biofilters and wetlands in three different climates

The following parameters were considered to be the key operational variables to be considered in defining the boundaries of validation:

1. Temperature, which is important for biodegradation in both biofilters [43] and wetlands [44], and
2. Length of dry periods (LDPs) between two events—e.g. it is reported that long dry periods are detrimental for nitrogen removal by biofilters [45], while very short dry periods are not desirable for pathogen removal [12] and micropollutant removal [13],
3. Volume of water that needs to be treated per event (along with the flow-rate this determines total detention time) is of importance for both wetlands [46] and biofilters [9],
4. Extreme wet conditions—it has been found that occurrence of two or more large consecutive events within short period can lead to breaking of the system function during the later events in which the system cannot provide reliable treatment [13],
5. Flow velocity through the wetland (or infiltration rate through the biofilter) is also crucial for any WSUD treatment system [46].

It should be noted that other variables, such as soil characteristics, are also important, but they are very system specific and their implications should be tested in Stage 2: Validation monitoring (Table 1).

The challenging values of the selected operational parameters, which are clearly dependent on climatic conditions, were determined for three different regions: humid sub-tropical (Brisbane, annual rainfall 1,000 mm); Mediterranean climate (Perth, annual rainfall 850 mm); and mild oceanic climate (Melbourne, annual rainfall 650 mm). They also had to be determined in relation to size and design of the WSUD systems (i.e. surface area, extended detention depth, permanent pool depth and outlet equivalent pipe diameter). The study followed the Australian standard design practice: i.e. biofilters are designed as per FAWB [47] guidelines (key design parameters are surface area, hydraulic conductivity, extended detention depth, filter depth and submerged zone) and wetlands as per current Melbourne Water design manual [48].

To determine (1) Challenge temperature, 30 years minimum and maximum daily temperature data from Bureau of Meteorology (BOM) (station No. 9225 in Perth, No.40245 in Brisbane and No. 86232 in Melbourne) were analysed by creating cumulative distribution curves. The extreme values (5^th^ percentiles of the minimum daily data as well the 95^th^ percentiles of the maximum daily data) were determined for each of the three climatic regions. 5^th^/95^th^ percentile is selected since it is usually acquired as the cut off in other validation procedures [14].

To determine the challenge values of (2) LDPs, (3) Volume per event, and (4) Extreme wet condition, the MUSIC V5.1 software package [49]—widely applied in Australian design practice [50]—was used. MUSIC was run for 384 selected designs of biofilters and 30 designs of wetlands for continuous 6 minute rainfall and monthly evaporation data measured between 1980 and 2010 in the three climatic regions. All these designs have covered the potential biofilter and wetland designs in real-life so that the work could be broadly applied. The detailed variable specifications for biofilter and wetland configurations in MUSIC are presented in S2 and S3 Tables, while the model parameters are summarized in S4 and S5 Tables. Properties of the catchment, and the link between catchment and treatment systems, are presented in S6 and S7 Tables.

All modelled events that produced outflows (over 30 years of continuous simulations) were used to construct probability cumulative curves of (i) LDPs and (ii) outflow volume per event for each examined design and climate type. These probability distribution functions (log-normal) were then applied to estimate the 95^th^ percentiles of the LDPs and volume of outflows, to determine their challenge values. The LDPs were determined as the duration between end and start of the outflow of two continuous events, which differs from the normal determination of LDPs (also called the Antecedent Dry Weather Period) that is based on the inflow. Statistics were formulated on outflows in this study because: (i) many events were either too small (having no outflow) or too large (leading to overflow), hence the inclusion of these events very likely resulting in significant errors if inflow was used in statistical analysis; and (ii) in terms of stormwater harvesting, treated water is more important; therefore to be on the safe side, the use of outflows for estimations was favoured. 1% of the maximum outflow-rate of the system (as a function of hydraulic conductivity, surface area, extended detention depth and filter depth; exclude overflow) was used as a cut-off to determine when outflow begins or ends. This cut-off value was determined with reference to experience from previous biofilter field experiments [13], in which it was established that when the flow-rate of outflow dropped to <1% of maximum flow-rate (i.e. 8.×10^–6^ m^3^/s), the measurement instrument could still provide the flow-rate value but no outflow could be observed at the outlet.

The fourth operational parameter, (4) Extreme wet condition, was defined as two consecutive events which are separated by a short dry weather period (i.e. a short LDP). There were two parameters that characterise such an event: (i) the LDP between the two consecutive events and (ii) the volume of stormwater treated in each consecutive event. To determine these parameters, a number of steps were followed:

- The LDP between every consecutive events which occurred in the 30 year rainfall period were calculated;
- All consecutive events which had LDPs greater than the 5^th^ percentile LDP were then removed from further analysis. This was to select only those pairs of consecutive events which were separated by small dry weather periods; and,
- The average volume of all remaining pairs of events was then calculated, and the 95^th^ percentile value was used as the challenge volume.

Biofilters and wetlands are designed to treat a certain capacity of rainfall event and a maximum design flow-rate above which inflow water is bypassed. Therefore the fifth operational parameter—(5) Maximum designed flow-rate—is always specified in the detailed system design for both biofilters and wetlands, hence should be directly adopted as the challenge flow-rate.

It should be acknowledged that the method presented here is quite flexible and subject to change. For example, other models could be used to simulate the hydraulic performance of the ‘to-be-validated’ system, such as Storm Water Management Model (SWMM), which is a dynamic hydrology-hydraulic water quantity and quality simulation model developed by USEPA [51] and the storage treatment overflow and runoff model (STORM) that is used to simulate the flow volume from watersheds, the bypass flow volume and the flow volume that passes through stormwater treatment systems [52]. Moreover, input data is not restricted to a 6 minute interval over 30 years; a minimum of 10 years of rainfall data, with relatively longer intervals, could be collected for statistical analysis.

## Results and Discussions

### Step 1: Target micropollutant in stormwater

Table 3 presents the estimated 5^th^, 50^th^ and 95^th^ percentile concentrations of 37 micropollutants in stormwater. The 95^th^ percentile concentrations of many micropollutants exceed drinking water guideline (DWG) values (Table 3). The estimated 95^th^ percentile concentrations of benzene (10μg/L) and benzo(a)pyrene (BaP) (8μg/L) were above all DWG values (i.e. 1 μg/L for benzene and 0.01 μg/L for BaP according to the most lenient guideline value in Australia). The 95^th^ percentile concentrations of di-(2-ethylhexyl)-phthalate (50μg/L) and pentachlorophenol (90μg/L) exceeded all DWG values except those for the EU which has not defined any guideline values for them. The estimated 95^th^ percentile concentrations of Aldrin and Dieldrin (2 μg/L and 0.2 μg/L respectively) were above Australian and WHO values (i.e. 0.3 μg/L for the sum of these two pesticides). EU DWGs has a maximum value of 0.1μg/L for an individual pesticide and 0.5μg/L for the sum of all pesticides. Hence, all 95^th^ percentile values for pesticides in Table 3 exceeded EU DWG values. Estimated 95^th^ percentile concentration of total PCB was 0.9μg/L, which is above the limit of USEPA guideline value (0.5μg/L). All such micropollutants detected above guideline values should be considered target micropollutants in stormwater. It is also recommended that site specific micropollutant data be used where available in preference to the adopted 95^th^ percentile values estimated in this study.

### Step 2: Treatment targets

The DWG values are considered as treatment targets: e.g. the challenge concentration of di-(2-ethylhexyl)-phthalate (DEHP) in stormwater was found to be 50 μg/L (Table 3); for potable reuse in Australia, the treatment system should be able to reduce the concentration to <10 μg/L, and this should be confirmed during validation monitoring in the second stage of the proposed validation framework (Table 1).

### Step 3: Potential removal mechanisms of micropollutants and the influential factors

Table 4 summarizes the physical-chemical properties of various micropollutant groups, potential dominating removal mechanisms, and the influential factors, which are based mainly on the study by Mackay *et al*. [38], Cottin *et al*. [23], Imfeld *et al*. [21], Abira M.A. *et al*. [24], Alvord *et al*. [25], DiBlasi *et al*. [27], V.H. Popov *et al*. [28],Larsen *et al*. [29] and Pavelic *et al*. [30]. In validation monitoring, identified potential mechanisms for a specific micropollutant or range of micropollutants will need to be validated separately.

**Table 4:** Physical-chemical properties of different micropollutant groups and their potential removal mechanisms and influential factors.

Micropollutants	Physical-chemical properties^#^			Potential dominating	Major influential factors
	Solubility [μg/L]	LogK_oc_	K_Henry_ [Pa·m^3^/mol]	mechanisms	
Herbicides	5.7×10^3^ ~9.0×10^5^	1.3–2.6	1.1×10^–5^ ~9.2×10^–4^	Adsorption*, biodegradation*, hydrolysis, photolysis	soil characteristics (organic content, nutrients), temperature, redox condition, etc
Phthalates	29~1.2×10^4^	2.7–5.0	0.004~3.2	Adsorption*, biodegradation	pH, temperature, soil characteristics, redox condition, etc.
Phenols	7.6×10^7^~9.3×10^7^	1.2–2.2	0.03~1.3	Biodegradation*, adsorption, volatilization	temperature, inflow concentration, retention time, redox condition, etc
Polychlorinated biphenyls	1.0×10^3^~1.6×10^5^	4.6–6.9	0.8~240	Adsorption*, biodegradation	soil characteristics, retention time, redox condition, etc
Halogenated aliphatics	1.4×10^5^~1.7×10^7^	1.5–2.7	7.7~540	Biodegradation*, volatilization*, adsorption	retention time, temperature, redox condition, etc
Monocyclic aromatics	1.3×10^5^–2.1×10^6^	1.1–3.0	270–1300	Biodegradation*, volatilization*, adsorption	retention time, temperature, redox condition, etc
PAHs	1.0~3.2×10^4^	3.1–7.4	0.009–43	Biodegradation*, adsorption*, volatilization	retention time, temperature, soil characteristics, etc

^#^ Data from Mackay *et al*. [38]

* the major removal process for the group

### Step 4: Potential surrogate parameters for micropollutant

A suite of readily measurable surrogates for organic micropollutants during wastewater treatment systems, such as aquifer recharge systems, chemical oxidation, and ozonation, was identified by Drewes *et al*. [39], Dickenson *et al*. [40] and Gerrity *et al*. [42]. Surrogates, such as the change of ultraviolet absorbance (delta-UVA), dissolved organic carbon (delta-DOC), delta-ammonia, and delta-nitrate, were suitable for monitoring biodegradation while delta-UVA and 3-D fluorescence are effective surrogates for adsorption [39]. These can be considered potential surrogates and should be verified by monitoring. It should be acknowledged that the surrogates suitable for micropollutants in chemical oxidation and ozonation may not be transferable to natural systems, however at this pre-validation stage it is better to form a bigger list of potential surrogates to be identified in later stages. Moreover, a wider range of readily measurable stormwater parameters, e.g. phosphorus, turbidity and electric conductivity, are also recommended to be tested in the second stage of the proposed validation framework (Table 1), where the correlation between a surrogate and corresponding pollutants needs to be elaborated. The suitable surrogate needs to be removed by the same mechanisms, and the surrogates concentration has to be highly correlated to the change in micropollutant concentration.

### Step 5: Operational and challenge conditions for biofilters and wetlands in three different climates

#### Challenge temperature

The estimated challenge low temperature (5^th^ percentile) and challenge high temperatures (95^th^ percentile) are shown in Table 5. As it is difficult to control the exact temperature during a field challenge test, it is recommended that field challenge tests be performed both in winter at a temperature no higher than the 5^th^ percentile value (e.g. 1.8°C in Brisbane) and summer at a temperature no less than the 95^th^ percentile value (e.g. 34.3°C in Brisbane) to cover the extremes.

**Table 5:** Estimated temperature percentile values in different climates.

Climate	Temperature (°C)	
	5^th^	95^th^
Subtropical (Brisbane)	1.8	34
Mediterranean (Perth)	-2.7	32
Oceanic (Melbourne)	5.0	33

#### The length of dry periods (LDPs)

**Fig 2:**
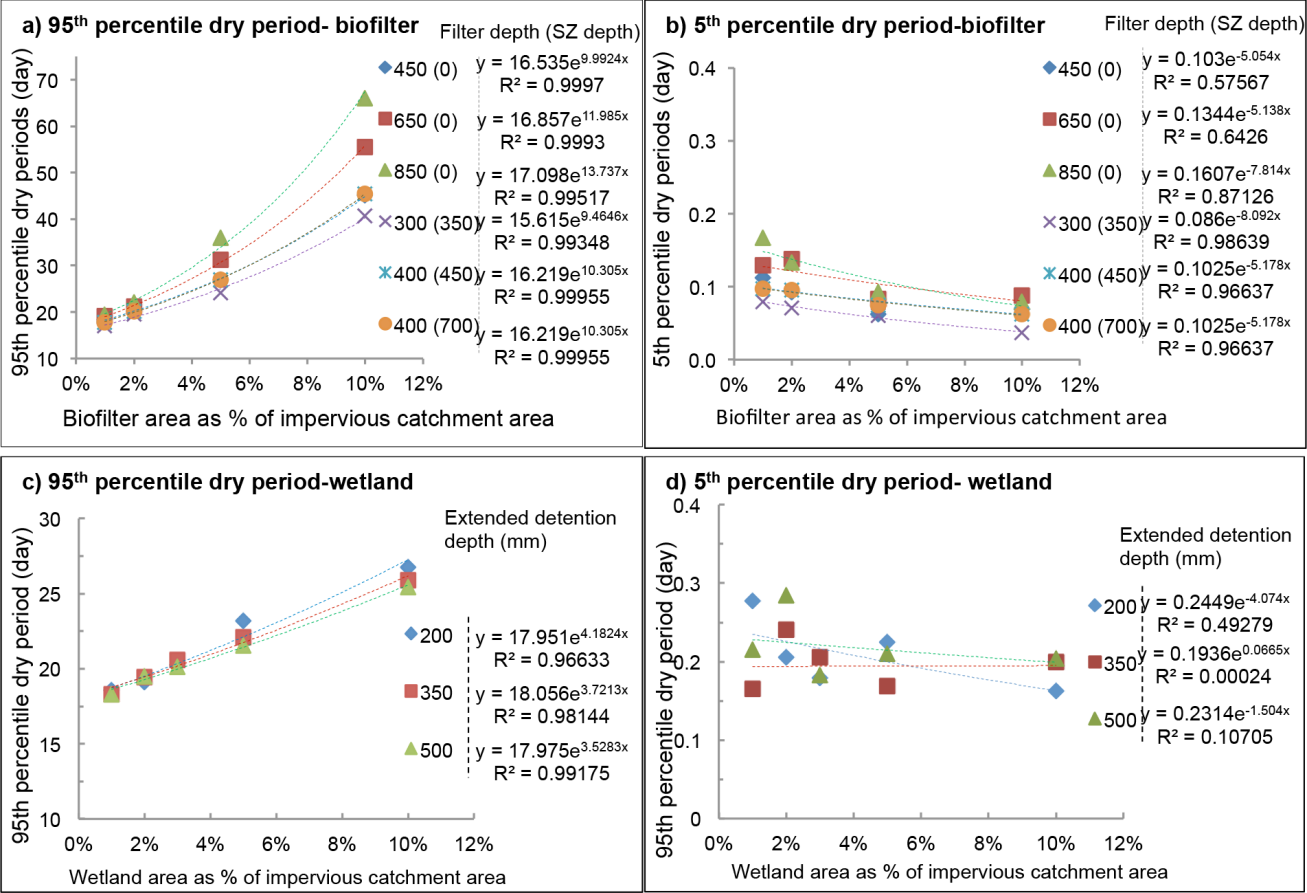
Plots of LDPs as a function of system area as percent of impervious catchment for mild oceanic climate (Melbourne): a) 95^th^ percentile and b) 5^th^ percentile dry period for biofilters at the designed hydraulic conductivity 100 mm/h and extended detention depth 200 mm. c) 95^th^ percentile and d) 5^th^ percentile dry periods for wetlands at permanent pool depth of 250mm. *SZ*: *submerged zone*.

Fig 2 shows example plots of the estimated LDPs as functions of the percentage of an impervious catchment area for biofilters and wetlands in Oceanic climate (Melbourne). The 95^th^ percentile LDPs are exponentially related to the system area (R^2^>0.90) for both systems and all climates. Other wetland design parameters had no observed relationship to LDPs (R^2^<0.05). For biofilters, LDPs increased with the increase of biofilter depth, and decreased with the increase in hydraulic conductivity. Extended detention depth of biofilters had no influence on the LDPs. Generally, if the system is larger in either size or depth, then smaller events would have no outflows and would be regarded as ‘dry’, hence the 95^th^ percentile of the LDPs of a larger system became higher. However, as for the 5^th^ percentile LDPs, the values decreased slightly alongside the increase of system size, whereas the difference between different system designs and types was relatively small, i.e. the 5^th^ percentile dry periods of all biofilters configurations were around 2 hours, whereas those of all wetland were 4–8 hours.

To further examine these results in relation to climate and size, for each systems size, the average 95^th^ and 5^th^ percentile of LDPs across all other system design parameters were calculated and presented in Table 6; this was undertaken since system size exerted the strongest impact on results. For the same design of biofilter or wetland area, the 95^th^ percentile LDP follows the order of Mediterranean (Perth)>Subtropical (Brisbane) >Oceanic (Melbourne) with an exception of Oceanic (Melbourne) being the longest at the largest biofilter area (10% of impervious catchment area).

**Table 6:** Estimated average values (±standard deviation) of different operational variable at different system areas.

System	City	Parameter		Unit	Biofilter/wetland area as % of catchment area				
					1.0%	2.0%	3.0% (wetland only)	5.0%	10.0%
Biofilter	Melbourne	Dry	95^th^	d	17±1.7	20±1.7	N/A	28±4.7	50±11
		period	5^th^	h	2.1±1.5	1.9±1.3	N/A	1.5±1.1	1.3±1.0
		95^th^	Single event	m^3^/m^2^	1.7±0.22	1.1±0.15	N/A	0.52±0.080	0.29±0.040
		volume	Consecutive event		1.2±0.29	0.75±0.18	N/A	0.40±0.12	0.17±0.060
	Perth	Dry	95^th^	d	26±6.9	28±7.1	N/A	34±11	41±13
		period	5^th^	h	1.8±1.6	1.7±1.4	N/A	1.3±1.1	1.0±0.80
		95^th^	Single event	m^3^/m^2^	2.8±0.53	1.7±0.42	N/A	0.77±0.24	0.38±0.12
		volume	Consecutive event		2.3±0.59	1.3±0.44	N/A	0.55±0.15	0.24±0.070
	Brisbane	Dry	95^th^	d	23±3.8	26±4.0	N/A	34±5.0	47±11
		period	5^th^	h	2.2±1.6	2.0±1.4	N/A	1.7±1.2	1.6±1.0
		95^th^	Single event	m^3^/m^2^	2.6±0.43	1.7±0.22	N/A	0.93±0.16	0.56±0.11
		volume	Consecutive event		2.2±0.96	1.4±0.61	N/A	0.72±0.22	0.41±0.11
Wetland	Melbourne	Dry	95^th^	d	18±0.14	19±0.16	20±0.20	22±0.75	26±0.61
		period	5^th^	h	5.3±1.2	5.8±0.86	4.5±0.31	4.8±0.64	4.5±0.46
		95^th^	Single event	m^3^/m^2^	1.8±0.59	1.4±0.36	1.0±0.17	0.71±0.080	0.38±0.040
		volume	Consecutive event		1.8±0.84	1.3±0.37	0.81±0.16	0.50±0.040	0.28±0.050
	Perth	Dry	95^th^	d	61±3.3	79±0.52	77±4.3	92±5.1	117±21
		period	5^th^	h	6.8±0.50	6.7±2.2	7.3±0.38	6.1±1.8	4.2±1.2
		95^th^	Single event	m^3^/m^2^	3.2±1.3	2.7±0.81	2.3±0.59	1.8±0.32	1.1±0.060
		volume	Consecutive event		3.1±1.8	2.6±0.43	2.4±0.96	1.5±0.25	0.94±0.16
	Brisbane	Dry	95^th^	d	31±0.65	35±0.39	36±0.26	37±0.12	48±35
	(HRT = 72h)	period	5^th^	h	7.4±0.96	7.6±1.2	6.3±0.35	6.6±0.60	5.6±0.13
		95^th^	Single event	m^3^/m^2^	1.4±0.56	1.2±0.46	1.0±0.31	0.84±0.24	0.63±0.13
		volume	Consecutive event		1.2±0.68	0.96±0.41	0.92±0.42	0.59±0.19	0.51±0.20
	Brisbane	Dry	95^th^	d	30±0.27	32±0.25	33±0.58	37±0.18	47±0.53
	(HRT = 48h)	period	5^th^	h	8.0±0.37	6.4±0.72	6.1±1.1	4.8±0.63	4.9±0.91
		95^th^	Single event	m^3^/m^2^	1.3±0.44	1.1±0.39	0.99±0.31	0.81±0.23	0.60±0.13
		volume	Consecutive event		0.77±0.20	0.88±0.26	0.75±0.20	0.60±0.13	0.43±0.10

#### Volume of water treated per event

**Fig 3:**
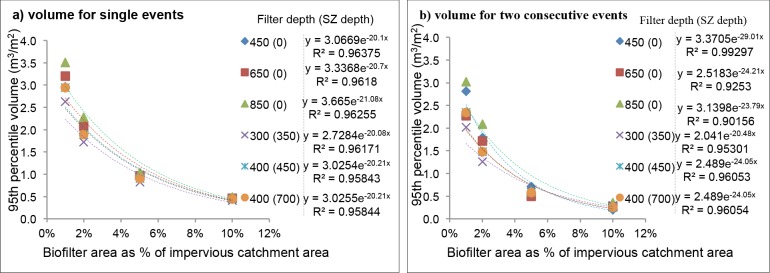
Plots of volume of water treated per area of biofilter as a function of biofilter area as percent of impervious catchment for Mediterranean climate (Perth) at the designed hydraulic conductivity 100 mm/h and extended detention depth 200 mm: a) 95^th^ percentile volume for single events and b) 95^th^ percentile volume for two consecutive events. *SZ: submerged zone*.

**Fig 4:**
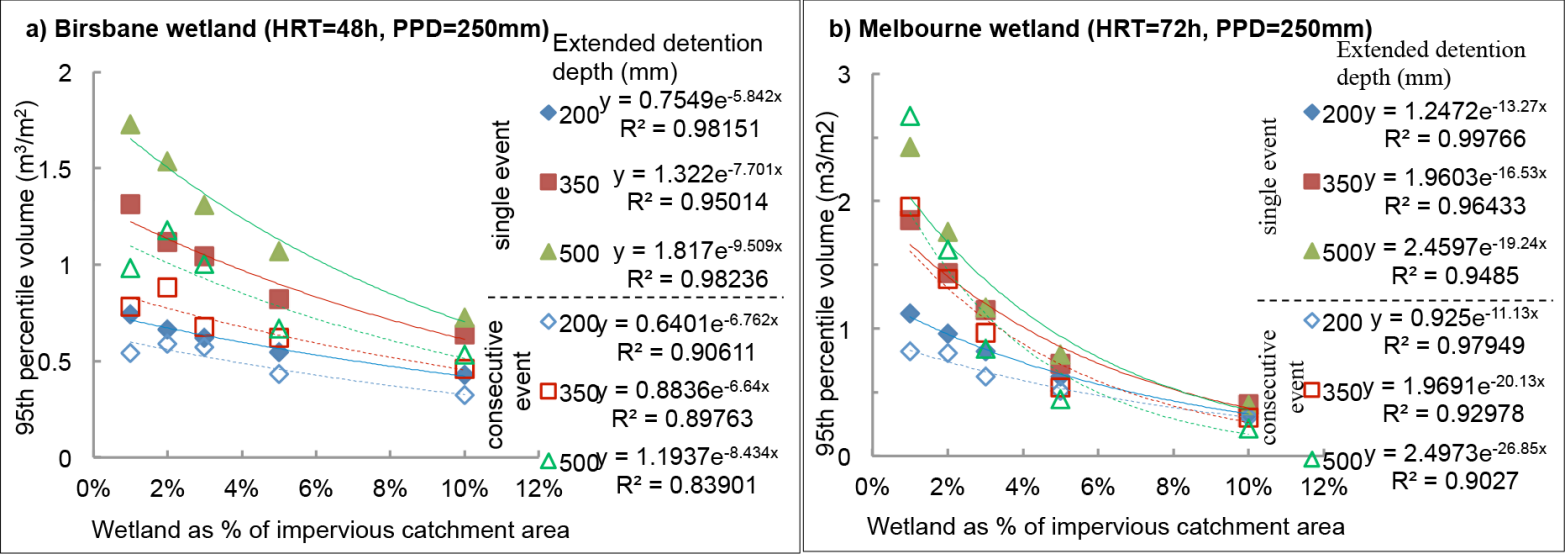
Plot of volume of water treated per unit area of wetland as a function of wetland area as percent of impervious catchment for: a) Brisbane wetland at the designed hydraulic resident time (HRT = 48 h) and permanent pool depth (PPD = 250 mm), and b) Melbourne wetland at the designed hydraulic resident time (HRT = 72 h) and permanent pool depth (PPD = 250 mm).

The 95^th^ percentile volumes per event were plotted against the system surface areas, and the exponential curves fitted are shown in Fig 3 for biofilters located in Mediterranean climate (Perth) and in Fig 4 for wetlands located in Subtropical (Brisbane) and Oceanic (Melbourne) climates.

Biofilters: Volume per area decreased with surface areas, filter depth and extended detention depth. The systems of equivalent total filter depth, but with and without submerged zone, had different treatment volumes; the systems without submerged zones were able to treat more water since they had higher hydraulic gradients. However, the depth of submerged zone had little relationship to the volumes, probably because the dry periods simulated were not long enough to dry the submerged zone.

The average volume for both single events and consecutive events was calculated for each system size and summarized in Table 6. Volumes for consecutive events were lower than that of one single event; the reason might be that the incidents of rainfall during consecutive events are more expansively spread. For a Mediterranean climate (Perth), the total average 95^th^ percentile volumes of consecutive events are about 74% of one single event, while percentages for oceanic (Melbourne) and subtropical (Brisbane) climates are 70% and 80% respectively. The 95^th^ percentile volume of either single event or consecutive events for biofilter follows the order of subtropical (Brisbane, 1008.2 mm/y)> Mediterranean (Perth, 850.0 mm/y)> oceanic (Melbourne, 649.6 mm/y) with an exception of Perth being the highest at the smallest biofilter design (1% of impervious catchment area) (Table 6).

Wetlands: The volume per unit area decreased with increasing surface area (Fig 4). In the majority of cases, the greater the extended detention depth, the greater the volume per area of water treated with the greatest difference in the smallest wetland areas. Permanent pool depth (PPD) had very little effect upon the volume treated per event.

The average 95^th^ percentile volume for both a single event and consecutive events for each wetland size appears in Table 6. Volumes for consecutive events are generally lower than that of a single event, with a few exceptions in small surface areas. Perth had the largest 95^th^ percentile volume of either single event or consecutive event, while Brisbane showed the smallest 95^th^ percentile volumes, except for the large surface areas, i.e. 5% and 10% of the impervious catchment area.

### Implications for the design of testings as Validation Monitoring

By applying the roadmap into the ‘to-be-validated’ system, all necessary information could be gathered through the five steps, and then proper validation monitoring testings can be designed. For example, the validation monitoring tests shall be performed in both winter (at temperature ≤5^th^ percentile value; Table 5) and summer time (at ≥95^th^ percentile value; Table 5) from Step 5; with determined 95^th^ percentile challenge volumes (Fig 3 for biofilter and Fig 4 for wetland; Step 5) of inflow water containing 95^th^ percentile challenge concentrations of target micropollutants (Table 3; Step 1) dosed into the ‘to-be-validated’ stormwater treatment systems. A series of testings is to be conducted to cover both challenge wet (5^th^ LDPs; Fig 2) and dry conditions (95^th^ LDPs; Fig 2; Step 5) to check if the treated water meets the treatment target (Step 2). Meanwhile, removal mechanisms of target micropollutants in Step 3 should be validated during *in-situ* tests (if possible) or laboratory studies. The identified potential surrogates (step 4) should be tested during validation monitoring to identify the most suitable surrogate parameters to be used in operational monitoring.

## Conclusions

If stormwater is to be treated for potable uses, Water Sensitive Urban Design (WSUD) should be used for preliminary treatment, and followed by advance treatment technologies to ensure required water standards are met. However, WSUD systems are the crucial step in such treatment trains, since they will reduce the high variability in stormwater quality and therefore ensure that the advance technologies perform well. One of the critical steps for using WSUD systems for safe stormwater harvesting is treatment validation. The successful validation of these systems will provide confidence to water regulators and the community at large, so that the treated stormwater can go directly to drinking water supplies.

The world’s first validation framework, consisting of three stages (i.e. pre-validation, validation monitoring and operational monitoring) for WSUD systems, was proposed. This paper focused on the pre-validation stage and developed a specific roadmap consisting of five steps with detailed methodologies: (1) identification of target micropollutants, (2) specification of treatment targets, (3) identification of potential removal mechanisms and influential factors, (4) identification of potential surrogates and (5) determination of operational and challenge conditions.

A literature search was undertaken to identify and quantify micropollutants in stormwater. Statistical analysis revealed that challenge concentrations of 8 pesticides, benzene, benzo(a)pyrene, pentachlorophenol (PCP), di-(2-ethylhexyl)-phthalate (DEHP) and total polychlorinated biphenyls (PCBs), were above different worldwide drinking water guideline limits, and hence set the treatment targets. Potential removal mechanisms (e.g. adsorption and biodegradation) for different micropollutant groups, as well as potential surrogates (e.g. delta-UV and delta-DOC), were identified through a literature review for further Validation monitoring and Operational monitoring.

A method that utilises MUSIC is suggested in this paper as a means of determining challenge conditions of stormwater treatment systems of varying design, using historical climate data. Results showed that 95^th^ percentile length of dry periods was exponentially related to system design area—as a percentage of an impervious catchment area—while the 5^th^ percentile length of dry periods remained within short durations (i.e. 2–8 hours). 95^th^ percentile volume of water treated per event was exponentially related to system design area as a percentage of an impervious catchment area.

## Supporting Information

S1 Table PRISMA Checklist. (XLSX)

S2 Table Raw data for analysis of micropollutant concentration in stormwater. (DOCX)

S3 Table Variable specifications for biofilter nodes in MUSIC. (DOCX)

S4 Table Variable specifications for wetland nodes in MUSIC. (DOCX)

S5 Table Fixed specifications for biofilter nodes in MUSIC. (DOCX)

S6 Table Fixed specifications for wetland nodes in MUSIC. (DOCX)

S7 Table Fixed specifications for catchment in MUSIC. (DOCX)

S8 Table Fixed specifications for drainage link/stormwater network pipe in MUSIC. (DOCX)
