# Supplementary material for: Methodologies for Pre-Validation of Biofilters and Wetlands for Stormwater Treatment
Source: PLoS One. 2015 May 8;10(5):e0125979. doi: 10.1371/journal.pone.0125979 (PMC4425486; doi:10.1371/journal.pone.0125979)
Supplement: S2 Table — (DOCX) [file pone.0125979.s002.docx]

**S2 Table. Variable specifications for biofilter nodes in MUSIC**

| Specification | Unit | Values | | | |  |
| --- | --- | --- | --- | --- | --- | --- |
| Surface area and Filter area | m^2^ | 100 | 200 | 500 | 1000 |  |
| Hydraulic conductivity | mm/hour | 50 | 100 | 300 | 600 |  |
| Extended detention depth | mm | 100 | 200 | 300 | 400 |  |
| Filter depth (submerged zone depth) | mm (mm) | 300 (350) | 400 (450 or 750) | 450 (0) | 650 (0) | 850 (0) |
| Note: if a specific system is give, then system specifications should be used. | | | | | | |
