# Supplementary material for: Methodologies for Pre-Validation of Biofilters and Wetlands for Stormwater Treatment
Source: PLoS One. 2015 May 8;10(5):e0125979. doi: 10.1371/journal.pone.0125979 (PMC4425486; doi:10.1371/journal.pone.0125979)
Supplement: S3 Table — (DOCX) [file pone.0125979.s003.docx]

**S3 Table. Variable specifications for wetland nodes in MUSIC**

| Specification | Unit | Values | | | | |
| --- | --- | --- | --- | --- | --- | --- |
| Surface area | m^2^ | 100 | 200 | 300 | 500 | 1000 |
| Inlet pond volume | m^3^ | calculated from: surface area×10% × inlet pond depth (1.5m) | | | | |
| Extended detention depth | mm | 200 | 350 | 500 |  | |
| Permanent pool depth | mm | 250 | 350 |  | | |
| Outlet equivalent pipe diameter | mm | * calculated from surface area, extended detention depth and theoretical detention time | | | | |
| Note: if a specific system is give, then system specifications should be used. | | | | | | |

* Equation$Q=Cc\frac{\pi D^{2}}{4}\sqrt{2gh}$, where Q=volumetric flow rate; Cc=0.6; D= equivalent pipe diameter; h=extended detention depth; and$Q=VA=\frac{h}{t} (0.895As)$, where t= theoretical detention time 72 h for all cities and 48 h only for Brisbane; A_S_=surface area of wetland.
