# Supplementary material for: Methodologies for Pre-Validation of Biofilters and Wetlands for Stormwater Treatment
Source: PLoS One. 2015 May 8;10(5):e0125979. doi: 10.1371/journal.pone.0125979 (PMC4425486; doi:10.1371/journal.pone.0125979)
Supplement: S4 Table — (DOCX) [file pone.0125979.s004.docx]

**S4 Table**. Fixed specifications for biofilter nodes in MUSIC

| Specification | Value | Comment |
| --- | --- | --- |
| Inlet properties (low flow bypass, high flow bypass) | Default  0 m^3^/s for low, and 100 m^3^/s for high | No low flow bypass for maximum harvest; high flow bypass as default to prevent scour from large storm events |
| Unlined filter media perimeter | 0.01 m | Minimum allowed by MUSIC (to avoid dividing by zero errors) |
| TN content of filter media / Orthophosphate content of filter media | Default – set for soil type: Sand | Not examined in this study |
| Exfiltration rate | 0 mm/hr | Biofilter fully lined for stormwater harvesting |
| Is base lined? | Yes | Biofilter fully lined for stormwater harvesting |
| Vegetation properties | Vegetated with effective nutrient removal plants | Nutrient removal plants make treatment for use more effective |
| Overflow weir width | 2 m | To be non-restrictive to flow |
| Underdrain present? | Yes | Drain present for harvesting, which means there is an outflow pipe collecting treated stormwater |
| Porosity of the filter media | 0.35 | Sand: 0.3 – 0.4 |
| Porosity of the submerged zone | 0.35 | Sand: 0.3 – 0.4 |
| Horizontal flow coefficient | 3 | Not important when the biofilter is lines/related to exfiltration through the sides of the biofilter |
| k-C* values for TSS, TN, TP | Default – set for soil type: Sand | Not examined in this study |
| Number of CSTR cells | 3 | Not examined in this study |
