# Supplementary material for: Methodologies for Pre-Validation of Biofilters and Wetlands for Stormwater Treatment
Source: PLoS One. 2015 May 8;10(5):e0125979. doi: 10.1371/journal.pone.0125979 (PMC4425486; doi:10.1371/journal.pone.0125979)
Supplement: S5 Table — (DOCX) [file pone.0125979.s005.docx]

**S5 Table. Fixed specifications for wetland nodes in MUSIC**

| Specification | Value | Comments |
| --- | --- | --- |
| Inlet properties (low flow by-pass, High flow by-pass) | Default  0 m^3^/s for low, and 100 m^3^/s for high; | Typical values required – not examined in this study |
| Storage properties: Vegetation cover (%) | Default | Typical values required – not examined in this study |
| Storage properties: Exfiltration rate | 0 mm/hr | Wetland analysed for stormwater harvesting |
| Storage properties: Evaporative loss as % of potential evapotranspiration (PET) | 125% | Typical value for study area; set to allow for transpiration through wetland vegetation as well as pond evaporation |
| Outlet properties: Overflow weir width | 3 m | To be non-restrictive to flow |
| Outlet properties: Notional detention time | 72 hours* | Recommended |
| Use custom outflow and storage relationship? | No | Not examined in this study |
| Orifice discharge coefficient | 0.6 | Default |
| Weir coefficient | 1.7 | Default |
| Number of CSTR cells | 4 | Not examined in this study |
| k-C values for TSS, TN, TP | Default for wetland | Not examined in this study |

*48 hours was also used for Brisbane.
