# Supplementary material for: Methodologies for Pre-Validation of Biofilters and Wetlands for Stormwater Treatment
Source: PLoS One. 2015 May 8;10(5):e0125979. doi: 10.1371/journal.pone.0125979 (PMC4425486; doi:10.1371/journal.pone.0125979)
Supplement: S6 Table — (DOCX) [file pone.0125979.s006.docx]

**S6 Table. Fixed specifications for catchment in MUSIC**

| Specification | Value | Comments |
| --- | --- | --- |
| Total area | 1 ha |  |
| Percent impervious | 100 | Surface runoff from impervious ONLY gets transferred to the treatment nodes |
| Impervious area properties – Rainfall threshold | 1 mm | Default value for runoff formation |
| Pervious area properties:  Soil storage capacity (mm)  Initial storage (% of capacity)  Field capacity (mm)  Infiltration capacity coefficient – a  Infiltration capacity exponent – b | Default:  120  30  80  200  1.0 | Not examined in this study |
| Groundwater properties  Initial depth (mm)  Daily recharge rate (%)  Daily baseflow rate (%)  Daily deep seepage rate (%) | Default:  10  25  5  0 | Not examined in this study |
