# Supplementary material for: Methodologies for Pre-Validation of Biofilters and Wetlands for Stormwater Treatment
Source: PLoS One. 2015 May 8;10(5):e0125979. doi: 10.1371/journal.pone.0125979 (PMC4425486; doi:10.1371/journal.pone.0125979)
Supplement: S7 Table — (DOCX) [file pone.0125979.s007.docx]

**S7 Table. Fixed specifications for drainage link/stormwater network pipe in MUSIC**

| Specification | Value | Comments |
| --- | --- | --- |
| Routing properties | No routing: Translation 0; Muskingum-Cunge not used | The pipe is considered to be SHORT, so there are no transformations to the stormwater hydrograph |
| Outflow components | Pipe flow; High flow bypass |  |
